# Sulphate of Quinine as an Excitant of the Uterus

**Published:** 1876-09

**Authors:** 


Sulphate of Quinine as an Excitant of the Uterus.—In a contribution to *La Presse Medicale Belge,* much testimony is brought forward in support of the power that sulphate of quinine possesses of awakening and exciting the action of the uterus. Dr. Paul reports two observations proving its efficacy for exciting the contractions of the fatigued uterus. Dr. Voghera says that quinine, given to some women who during pregnancy were attacked with neuralgia, or for other causes, provoked abortion; that, given to some women at the full term of pregnancy, it brought on labor; and that, when these women experienced slight irregular pains, the sulphate of quinine rapidly caused the expulsion of the fœtus. It likewise facilitates the expulsion of the placenta, by exciting uterine contractions; and when in the puerperal state the lochia are suspended, a dose of quinine is sufficient to re-establish them. He likewise states that in some rare cases the prolonged use of quinine suppressed the lacteal secretion, and brought on the menstrual flux. Dr. Ombini Vincent relates two cases of difficult labor from inertia of the uterus, and two cases of metrorrhagia, that were overcome by quinine. Dr. Louis Aporti declares to have found in it a more prompt and energetic action than in ergot. Dr. Losi Carlo likewise congratulates himself on having substituted quinine for ergot, and asserts to have found it a powerful medicine either to awaken suspended contractions, or to strengthen them where they were weakened. Dr. Bouque recounted a very interesting observation of a case of metrorrhagia, rebellious to every other treatment than quinine. He admits that quinine is endowed with excito-motor powers over the vaso-motor nerves, and in this manner explains its hemostatic power in every case where contraction of the capillaries is insufficient or wanting.—*The Doctor.*

